# #ButNotMaternity: Analysing Instagram posts of reproductive politics under pandemic crisis

**DOI:** 10.1177/13675494231173661

**Published:** 2023-05-28

**Authors:** Sara De Benedictis, Kaitlynn Mendes

**Affiliations:** Brunel University London, UK; Western University, Canada

**Keywords:** #ButNotMaternity, COVID-19 pandemic, digital feminist activism, freelance feminism, Instagram, maternity, reproductive politics

## Abstract

In this article, we perform a thematic analysis of a sample of 70 #ButNotMaternity Instagram posts. #ButNotMaternity is a hashtag that emerged in the United Kingdom during the COVID-19 pandemic whereby the public, healthcare workers and campaigners shared experiences and concerns about pandemic maternity care restrictions and their disproportionate disadvantages for pregnant women. In the article, we analyse four themes that emerged from our thematic analysis – Individual experiences, loneliness and overcoming adversity, Voicing anger and absurdity, Mobilising anger and calls to action and Coordinated activism. Thinking about #ButNotMaternity in the context of ‘freelance feminism’, our article has a twofold aim. First, we explore the concept of ‘freelance feminism’ through #ButNotMaternity, asking to what extent this campaign draws from freelance tactics. Second, we use the hashtag to illuminate maternity inequality and modes of resistance during the COVID-19 pandemic. Through our thematic analysis, we argue that while ‘freelance feminism’ might be becoming hegemonic as a dominant mode of organising feminist activism and resistance, inspired by Malik et al. (2020), we also showcase how creative campaigns are potential places where collective action, structural critique and resistance may emerge.

## Introduction

The COVID-19 lockdown measures beginning in March 2020 saw UK pregnancy and maternity inequality highlighted in the context of the pandemic. Despite the World Health Organization (WHO, 2020) stating that those giving birth in hospital should be allowed birthing partners throughout the pandemic, UK lockdown restrictions tightened in September 2020. As Menzel (2022: 84) notes, these policies effectively ‘“barred” many expectant fathers in the UK [. . .] from maternity care spaces meaning that they may have “missed out” on important milestone appointments, sometimes even the birth, of their child’. As this situation unfolded, doulas (birth companions) from The BirthBliss Academy launched #ButNotMaternity. Under this hashtag, the public, healthcare workers and campaigners shared experiences and concerns about pandemic maternity care restrictions and their disproportionate disadvantages for pregnant women. This campaign, and the subsequently formed But Not Maternity Alliance,^1^ saw experiences shared, #ButNotMaternity scrawled on celebrities’ pregnant bumps, companies like *Vogue* supporting the cause, 644,585 petition signatures by January 2022 (Change.org, 2022) and open letters signed by MPs. This paved the way for policy changes from the Scottish Government, NHS England and more (But Not Maternity, 2022). The success of the campaign, which draws on various tactics to demand change, has been attributed to good research, case studies, political links, social media pressure and, crucially, mainstream media coverage (Forest and Popplewell, 2021). Moreover, although the majority of lockdown restrictions have ended, as recently as November 2021 institutions continued to face legal challenges to maternity restrictions from some organisations within the But Not Maternity alliance (Birthrights, 2021). As such, #ButNotMaternity offers accounts of maternity inequality within a context of a pandemic crisis, illuminating how pregnant women, activists and feminist campaigning groups have resisted this inequality and influenced political decisions (see also Menzel, 2022). We can also regard #ButNotMaternity both as part of a broader re/invigoration of reproductive politics (De Benedictis, 2022; Franklin and Ginsburg, 2019) emerging within a period of pandemic and welfare crisis (see Patrick et al., 2022), and a rise in mainstream feminism (Banet-Weiser, 2018; Rottenberg, 2018).

As this Special Issue attests, feminism is not only becoming mainstream in the cultural sphere, but is increasingly ‘freelanced’, whereby ‘feminist projects are now working through a casualised gig economy’ in which ‘models of entrepreneurial precarious labour [. . .] are being used to sustain feminist work’ (Curran-Troop et al., Forthcoming). The conditions of ‘freelance feminism’ may be shaping, and restraining, the types of feminist work that is able to exist in the public sphere, moving towards an emphasis on individuals withstanding these precarious contexts rather than forming collective action. Questions therefore are raised about how a campaign such as #ButNotMaternity – which draws on both freelance traits, such as (unpaid) digital activist labour, and more traditional forms of (paid) institutionalised feminist activism, such as campaigning and non-governmental organisation–led research – play out in such a context? Thinking about #ButNotMaternity in this context, our article has a twofold aim. First, we explore the concept of ‘freelance feminism’ through #ButNotMaternity, asking to what extent this campaign draws from freelance tactics. Second, we use the hashtag to illuminate maternity inequality and modes of resistance during the COVID-19 pandemic. Through a thematic analysis of Instagram posts using the #ButNotMaternity hashtag, we argue that while ‘freelance feminism’ might be becoming hegemonic as a dominant mode of organising feminist activism and resistance, creative campaigns that ‘seek to “talk back”, to reimagine and reign in powerful forms of oppression’ (Malik et al., 2020: 9) are also potential places where collective action, structural critique and potential resistance may emerge. Malik et al. (2020: 9) demonstrated this in relation to resistant creativity from organisations and groups fighting back against racism and other forms of prejudice. Indeed, through analysing four key themes that emerged within #ButNotMaternity posts, we show that the principles of entrepreneurialism, market competition, and insecure and precarious labour are driving principles within mainstream feminism, yet these exist alongside potentially alternative framings. We conclude this article by offering some preliminary thoughts about why it might be that #ButNotMaternity does not fully follow the ideological framings offered by recent accounts of ‘freelance feminism’.

## Pandemic maternity inequality and feminist digital activism

### Pregnancy and maternity inequality

For some time, scholars and activists have documented how pregnancy and maternity have been shaped by broader neoliberal shifts that have governed various Western nations. As maternity has become increasingly visible in the public sphere (Tyler, 2011), scholars have illuminated how notions of choice (Orgad, 2019), surveillance (Tyler, 2011) and confidence (Orgad and Gill, 2022) that have characterised broader feminine subjectivities under neoliberalism have also shaped maternal subjectivities in the sociocultural realm. Within such neoliberal formations, those that are marked as ‘good’ maternal subjects are often white, heterosexual and middle-class (e.g. Allen et al., 2015; Jensen, 2018; Orgad and De Benedictis, 2015). While such ‘maternal publicity’ (Tyler, 2011) reigns, inequality related to pregnancy and maternity continues to mark maternal experiences in the United Kingdom (Casey et al., 2022; Menzel, 2022). Indeed, scholars have noted how racism is ‘at the root’ of many inequities in UK maternity care, resulting in significantly higher mortality rates for Black women and their babies (see Waters, 2022). Beyond adverse birth outcomes, others, such as Orgad (2019: 43), note that the ‘motherhood penalty’ shapes women’s earnings, and pregnancy discrimination sees 60,000 women lose their job each year (Brearley cited in Orgad, 2019: 43).

While inequity and discrimination are long-standing issues for new and expecting mothers, the pandemic brought pregnancy and maternity into sharp focus. Although academic research on maternity and pregnancy experiences within the pandemic is limited (with exceptions)^2^, some organisations, charity groups and research institutes have conducted their own research. The United Nations (2020) warn of stark global inequities due to the COVID-19 pandemic, estimating a 38 percent maternal mortality increase due to health system disruption and worsening of maternal and child health outcomes, and others (e.g. Fernandez Turienzo et al., 2021) note that these inequalities may be particularly felt by Global Majority people and groups. At the beginning of the pandemic in the UK, the impact of COVID-19 to pregnancy was unknown and a blanket ‘stay-at-home’ order was issued to pregnant people. As the pandemic went on, as some sectors began to feel the impact of the pandemic, and lockdown restrictions saw workers furloughed, made redundant and children having to stay at home, surveys showed that women felt they were made redundant (Pregnant Then Screwed, 2020) and could not accept work in some industries (e.g. television) due to childcare issues (Wreyford et al., 2021). In terms of working conditions, 45 percent of pregnant women who were working outside of the home had not had a risk assessment and this increased to 52 percent for Global majority women (Pregnant Then Screwed, 2020). One area in which pregnancy discrimination during the pandemic occupied a particularly public lens was in relation to birthing. Here, as women faced birthing alone, social media became a space in which people voiced their experiences and protested these conditions (see also Menzel, 2022).

### Digital feminist activism and maternity

In recent years, scholars have explored the uptake of digital technologies and social media platforms to ‘dialogue, network, organise and challenge’ (Mendes et al., 2019b: 2) various systems of oppression linked to patriarchy, racism, capitalism and more (Fotopoulou, 2016; see Clark-Parsons, 2018). This work notes the ways feminists increasingly draw on networked media, including hashtags, to accomplish their goals (Clark-Parsons, 2018; Jackson et al., 2020; Loza, 2014). Although much research on digital feminist activism focuses on issues such as gender-based and sexual violence (see Dey, 2018; Jackson et al., 2020; Loney-Howes, 2020; Mendes, 2015), others have also explored issues around maternity inequality and care they received, and the ways they have harnessed social media to make these visible (van der Pijl et al., 2020). Van der Pijl et al. (2020), for example, studied the Dutch hashtag #genoeggezwegen (#breakthesilence) to explore how women were sharing negative and traumatic maternity care experiences.

The strategic use of the hashtag has been credited for helping to secure visibility, recognition and attention to issues if enough people mobilise around it (see Jackson et al., 2020; Williams, 2015). This is often, though not exclusively, done through individual narratives which, when read collectively in the context of feminist activism, demonstrate how personal experiences can be both deeply political (see Boyle, 2019) and highly *affective* (see Mendes et al., 2018). Indeed, the affective turn in scholarship (Karatzogianni and Kuntsman, 2012) has been crucial for scholars theorising why certain movements or campaigns are more successful than others, and how individual and collective narratives build affective solidarity and publics capable of shifting consciousness, policies and practices (Hemmings, 2012; Nau et al., 2022; Papacharissi, 2015).

There have been many real and valid criticisms of the limits of digital feminist activism, particularly for its focus on the experiences of white, Western, middle-class, able-bodied, cis-het women (e.g. Boyle, 2019; Gill and Orgad, 2018b; Onwuachi-Willig, 2018; Phipps, 2020; Salvatori and Mendes, 2022), yet it is also important not to discount their actualised and potential impact on society. Indeed, contemporary scholars have complicated ‘ideas that social media encourages fantasies of individual change rather than genuine material transformations and activism’ (Mendes et al., 2019b: 177), and have pointed out how digital feminist activism can and has led to material changes – in laws, policies, practices, understanding and beliefs (see Boyle and Cucchiara, 2018; Chandra and Erlingsdóttir, 2020; Jackson et al., 2020; Levy and Mattsson, 2019; Salvatori and Mendes, 2022; Szekeres et al., 2020). It is with this framing in mind, and the understanding that many of these changes, particularly cultural ones, are difficult to measure, that we explore #ButNotMaternity, and its (im)measurable outcomes and potential.

## Methodology

To analyse the nuances of the #ButNotMaternity campaign, we undertook a thematic analysis (Braun and Clarke, 2006) of Instagram posts on the campaign hashtag. We began by doing a preliminary investigation of the representation of #ButNotMaternity in mainstream news sites and blogs, which often upheld the spectacle of the pregnant body (Tyler, 2011) to discuss the campaign. Due to the visual nature of the campaign in wider news, from there we decided that analysing Instagram posts of the campaign would provide insight into its visual and cultural impact.

Following other studies of Instagram, we aimed to undertake a digital ethnography of social media (Postill and Pink, 2012), taking an ‘iterative, hermeneutical approach’ to analysing the texts (Drenten et al., 2020: 49). #ButNotMaternity is a publicly open hashtag and only posts by those with publicly open accounts were visible. In 2022, De Benedictis searched the campaign hashtag on the Instagram application and from the ‘Top Posts’ section she collected posts that used #ButNotMaternity in the description or tagged in the photo. If posts were irrelevant or duplicates, then they were not collected. Videos were also not analysed. De Benedictis collected 70 ‘Top Posts’, dating from 2 September 2020 to 29 June 2022. Based on previous research conducted by Mendes and others, it was determined this initial sample size would be large enough to reach saturation (Mendes et al., 2019b). The posts were recorded using the screenshot function, and if a post had multiple images, then screenshots would be taken of all of them. This resulted in 114 images and related captions from 70 posts for us to analyse. We used an iterative approach where both authors began by analysing images independently. In consultation with the key literature, we compiled 24 subthemes across the dataset and organised those that featured the most into four main themes. We began by jointly coding half the images, and independently coding the other half, once we were certain we had intercoder reliability. De Benedictis then checked and finetuned all the themes in the items.

As we analysed ‘Top Posts’, we included ‘the visual content (e.g. image), the text (e.g. captions and hashtags) and interactive affordances (e.g. likes and comments) of each post’ (Drenten et al., 2020: 49). However, it became clear as we undertook this process that some posts did not have as many likes as others, suggesting that they may not have been ‘Top Posts’, which we suspect is because Instagram removes posts on ‘Top’ or ‘Recent’ posts that violate their Community Guidelines (Instagram, n.d.). While Instagram releases few details about its algorithmic architecture, it is important to recognise that algorithms play an important role in structuring our online experiences (see Cotter, 2019). For researchers, this also means the algorithm and API also have an impact on our sample, and we are aware that the sample will likely change for other scholars wishing to replicate this study. Following best practice guidelines of social media research (Ahmed et al., 2017), we have not referenced the Instagram users’ handle, directly quoted from their posts or replicated their images. We begin below with the first of our four key themes – a focus on individual experiences, loneliness and overcoming adversity.

## Analysing #ButNotMaternity on Instagram

### Individual experiences, loneliness and overcoming adversity

The thematic analysis of the #ButNotMaternity Instagram posts illuminated some individualising registers that we might expect to see within a context of ‘freelance feminism’, which is shaped by neoliberal (Rottenberg, 2018) and popular feminism (Banet-Weiser, 2018). Many of the Instagram posts detailed the individual experiences of pregnant women who had given birth during the pandemic. Narratives of pregnant women waiting in wards alone, getting news about their pregnancies without support and birthing in isolation emerged through the #ButNotMaternity posts. The underlying mood of many of these posts that detailed individual experiences of pregnant women was that giving birth under lockdown restrictions gave way to loneliness, isolation and anxiety. Some of the posts pictured an eerily empty ward as the pregnant woman went in to give birth, a visual contradiction to the discourse of the overwhelmed and overcrowded NHS that has marked the public imagination of the NHS for some time (e.g. Puttick, 2017; Wooler, 2018). Other posts stressed that giving birth in such isolating conditions without a birth partner increased levels of anxiety and worsened mental health conditions. Such loneliness and isolation was often attributed to not having a male partner with them at birth, not having a birthing advocate and missing out on bonding with other pregnant women (see also Menzel, 2022). In this sense, we can see that maternity inequality was predominately framed as an issue affecting cis-heterosexual women and their partners.^3^ This finding supports broader representations of birth in mainstream media (De Benedictis et al., 2019).

We found many posts were laden with affective registers, with confessions or statements about how (expectant) mothers were suffused with guilt for not being able to give their child the start in life that they wanted. This was due to lockdown restrictions shaping maternity care, extending beyond this to missing out on playgroups and other baby classes. Such maternal guilt can be understood in relation to ideas about the ‘good enough’ mother. Lawler (2000) argues that sociocultural constructions of the ‘good enough’ mother uphold mothers as guarantors of the social world with an underlying assumption ‘that mothers produce (gendered) selves; that the social world could be transformed through the transformation of these selves; and, therefore, that it is mothering which can (and should) transform the social world’ (p. 51). Within the context of the pandemic, due to the long-standing ‘good enough’ mother discourse, mothers arguably symbolise an important role in alleviating the pandemic’s social crisis through taking on home care, schooling and much more unpaid work to weather the storm. This is particularly the case for families that were increasingly neglected within a withering welfare state (Orgad and Gill, 2022: 104). Yet, simultaneously, maintaining such ideas about the ‘good enough’ mother becomes increasingly fraught as mothers could not do the things that they are usually encouraged to do to nurture their children in pre-pandemic times through the middle-class intensive mothering discourse (see Jensen, 2018).

Supporting this theme, we found many discourses across #ButNotMaternity posts that stressed that within the pandemic context people experiencing pregnancy and birth needed to ‘keep calm and carry on’. Although this British wartime mantra was revitalised after the 2008 economic recession, it is part of a growing ‘resilience discourse’ (Swan, 2017) steeped in neoliberalism that prioritises individuals overcoming structural inequalities and forms of oppression (see Bull and Allen, 2018; Gill and Orgad, 2018a). Some posts stressed that to overcome the adversity of their situation, they needed to have a ‘positive’ and ‘calm’ mindset, with some posts going as far as to offer advice. This ranged from using FaceTime or video scans when birth partners could not join (see also Menzel, 2022), recording baby’s heartbeat to share at a later date, using hypnobirthing to keep a positive mindset and staying home as long as one could during labour. Other posts were community-minded and meant to support pregnant women. This included those that congratulated ‘super mums’ who were giving birth under lockdown restrictions and generally celebrated those who, through individual effort, grit and a positive mindset, overcame structural barriers imposed by the state. These posts often stressed that one day they would look back and be proud that they managed to get through this highly unusual situation.

The positive framing of these pandemic experiences within the posts chimes with broader ideas about what Orgad and Gill (2022) term ‘confident mothering’. Orgad and Gill (2022) argue that ‘confident mothering’ is increasingly prevalent in sociocultural sites whereby mothers are put forward as ‘subjects that need to transform themselves and their daughters from insecure subjects in crisis to confident and resilient women and girls’ as the state further withdraws from offering families support (Orgad and Gill, 2022: 105; see also Jensen, 2018). They note that under ‘confident mothering’, positive affects are key to instilling an ‘authentic’ confident mentality, and negative emotions are often silenced and suppressed (Orgad and Gill, 2022: 110–111). Within posts on #ButNotMaternity, therefore, confidence and a resilient spirit were offered as individualised ways to overcome the difficult structural maternity experiences under lockdown. These discourses adhere to characteristics of ‘freelance feminism’, in that unpaid digital labour and individualised confessions and insights made up a substantial amount of the hashtag’s presence on Instagram.

### Voicing anger and absurdity

While such upbeat affect marked many #ButNotMaternity posts on Instagram, we also witnessed an overwhelming mood of anger expressed through the posts. These posts potentially stand in stark contrast to broader discourses about ‘confident mothering’ due to the activist nature of the #ButNotMaternity campaign. The presence of anger and other negative affective registers such as sadness has been documented by others examining social media activism (Nau et al., 2022) and supports scholars’ arguments that in a post #MeToo era, rage and anger are increasingly visible in contemporary gender representations (Kay, 2020; Kay and Banet-Weiser, 2019; Orgad and Gill, 2019). Many of the posts on #ButNotMaternity voiced individualised anger about maternity experiences during lockdown and the unfairness of the situation that mothers and pregnant women found themselves in.

At first glance, many of these posts enticed users in through imagery conveying happiness about their upcoming or new arrival. This included aesthetically pleasing shots of baby bumps, a happy couple or a baby shot that is familiar within the neoliberal aesthetic and regulation of ‘intensive pregnancy’ on Instagram (Tiidenberg and Baym, 2017). Here, pregnancy is positioned through responsibility, labour and consumerism, particularly through the lens of the sexualised pregnant body (Tiidenberg and Baym, 2017). However, a closer look at the posts’ captions reveals expressions of anger at having to birth alone, lockdown restrictions and the Conservative government control of these, as we elaborate below. Like ‘pregnant stand-up’ performances, the images, therefore, juxtapose with the written text in an incongruous manner to say something different about maternity within the pandemic context (see Lockyer and De Benedictis, 2023). In this sense, we can see that the posts have a dual purpose. On the one hand, they highlight the myth of pregnancy on Instagram by underscoring the curated nature of such posts. On the other hand, they use familiar pregnancy conventions to say something new about pandemic maternity inequality while disrupting Instagram’s normative expectations of pregnancy (see Lockyer and De Benedictis, 2023). Therefore, these posts could be seen to use Barthes’ notion of ‘counter myth’. As Barthes notes, the ‘[b]est weapon against myth is perhaps to mystify it in its turn: and to produce an artificial myth and this reconstituted myth will in fact be mythology’ (Barthes, 1972: 135).

What is striking about the prevalence of anger in this example of feminist activism is how it has been used as a device not to uphold the status quo, as we might expect in the context of entrepreneurial, or ‘freelance feminism’, but to mobilise others and call for systemic change.

### Mobilising anger and calls to action

Anger, as we noted above, was not only individualised within #ButNotMaternity to express discontent with the poster’s personal circumstances. In many posts, anger was targeted and used to critique government policies and politicians. For example, posts created during the most intense periods of lockdown restriction often emphasised the absurdity of the current maternity restriction policies. They often did this by comparing them to other restrictions that had been lifted. Images and captions would often discuss the lifting of leisure restrictions, such as being able to go to the pub or cinema, while maternity restrictions remained (see also Menzel, 2022). In *The Cultural Politics of Emotion*, Ahmed (2004) analyses the significance of anger to feminist movements, noting that ‘[a]nger is creative; it works to create a language with which to respond to that which one is against, whereby “the what” is renamed, and brought into a feminist world’ (Ahmed, 2004: 176). Within #ButNotMaternity, the public used anger to critique structural inequities and align themselves together as a collective. Drawing on Ahmed, we can see some posters were renaming ‘the what’ by directly calling the maternity lockdown restrictions a silencing of women, an impingement on human rights and an infringement on birth rights.

Another flashpoint in which anger was expressed in #ButNotMaternity posts was in response to #partygate. Beginning in the spring of 2022, a stream of leaked videos, images and reports emerged dating back to November 2021, in which civil servants and senior politicians flouted their own lockdown restrictions, socialised together and held parties as the United Kingdom was in the depths of the COVID-19 pandemic (BBC News, 2022a). An inquiry was launched and resulted in a damning report from the senior civil servant, Sue Gray. The Metropolitan Police subsequently issued 126 fixed penalty notices for breaking the lockdown laws, including to the then Prime Minister, Boris Johnson, and Chancellor, Rishi Sunak (see BBC News, 2022b), and the scandal ultimately saw Johnson resign from his role.

The #ButNotMaternity posts that referenced this scandal did so at the key moments in which news about the scandal broke, reports were released and public opinion was turning against Johnson after many lockdown restrictions had been lifted. Many posts contrasted their own experiences of giving birth in isolation and difficult circumstances with what politicians were doing at the time, such as having secret parties or affairs (referencing the affair of the then Health Secretary, Matt Hancock, with his aide, Gina Coladangelo). These individual and highly affective experiences, when read together, tell a powerful story of the broken system of the British government at this time, simultaneously calling for changes to be made. Scholars have documented how digital campaigns (e.g. #MeToo) can voice marginalised experiences, spark global conversations and lead to policy change (Jackson et al., 2020; Loney-Howes et al., 2021; Mendes et al., 2019a). We read the #ButNotMaternity hashtag, and particularly posts in the wake of #partygate, as contributing to the wider public conversation and public pressure that led to the downfall of Boris Johnson as Prime Minister. As Ahmed (2004) notes,

it is not just pain that compels us to move into feminism – or compels feminism as a movement of social and political transformation. The response to pain, as a call to action, also requires anger; an interpretation that pain is wrong, that it is an outrage, and that something must be done about it. (p. 174)

### Coordinated activism

It was not just during #partygate that we saw posters demanding change or calling others to action. Indeed, at times these calls to action were much more highly coordinated and centralised. During lockdown, charities and advocacy groups used the #ButNotMaternity hashtag in direct calls to action, imploring members of the public to act. They did this in various ways, by asking them to contact hospital trusts and MPs to support the #ButNotMaternity campaign, to fill out surveys, sign petitions, share research and information about the legal stance of the maternity restrictions, raise awareness of the issue by taking photographs supporting #ButNotMaternity and by sharing other people’s photos supporting the campaign. After the #ButNotMaternity campaign was initially launched, and the subsequently formed But Not Maternity (2022) Alliance came together, policy changes from the Royal College of Midwives, the Scottish Government, Northern Ireland’s Department of Health and NHS England were soon to follow. Some #ButNotMaternity member organisations (Pregnant then Screwed and Better Births) formed the Lone Births Campaign with the *Mail on Sunday*, headed by MP Alicia Kearns, and received backing from the Health Secretary and Prime Minister, resulting in change to NHS guidance. In this sense, we can see that the #ButNotMaternity hashtag was part of a larger, more traditionally organised campaign to directly change maternity restrictions. Indeed, some of the posts in our sample served to celebrate and mark these changes and the campaigns’ success. When thinking about #ButNotMaternity in the context of ‘freelance feminism’, the hashtag, we argue, exemplifies a combination of more traditional forms of (feminist) organising in the charity sector, as well as newer forms of digital feminist activism that we have seen emerge within what some have termed the fourth wave of feminism (Munro, 2013; Rivers, 2017). Rather than solely relying on the unpaid labour of everyday citizens or activists, #ButNotMaternity uses a combination of precarious entrepreneurial labour alongside work carried out by established organisations, supported by paid staff. As such #ButNotMaternity paints a complicated picture of ‘freelance feminism’ as a dominant mode of feminist organising, activism and discourse.

## Conclusion

In this article, we have argued that the social media campaign, #ButNotMaternity, highlights how the new terrain of ‘freelance feminism’ is a complex rather than simplistic one. While it is certainly true that ‘freelance feminism’ is becoming a dominant mode of feminist organising, we showcase its complexity in relation to digital feminist activism and campaigning. Our article highlighted four themes that arose from an analysis of the #ButNotMaternity hashtag. These were: individualised narratives of loneliness in which posters stressed overcoming adversity in the context of lockdown maternity restrictions; individualised formations of anger and rage about the experiences of lockdown maternity restrictions aimed at undefined actors that resented the absurdity of the situation; mobilised and collective forms of anger; and coordinated activism that was aimed at politicians, government policies and the government more generally, which was often linked to a call to action from feminist campaigners for social media users to act in specific ways to apply pressure for those in power to cease maternity lockdown restrictions. We can see, therefore, that the #ButNotMaternity campaign follows some of the key features of ‘freelance feminism’. The campaign evidences how individuals are called upon to engage in digital labour through hashtag activism. As others have shown, digital feminist labour is highly precarious and affective (see Jarrett, 2016; Mendes, 2022). However, there are elements of the campaign that do not fit within this framing of ‘freelance feminism’. Such features include the way the campaign was organised by those with paid positions (e.g. doulas, charity sector workers). It also includes the way the campaign calls for structural change – in terms of policies, practices and even political leadership. While we see a general move for feminism in the mainstream to be increasingly organised by the principles of entrepreneurialism, market competition and insecure and precarious labour, there still are outliers to this dominant framing. And although these outliers may be defined by the same key features of ‘freelance feminism’, they nonetheless need our attention as they may offer alternative narratives to its mainstream story.

So why is it that #ButNotMaternity does not fully follow the mainstream and dominant formations of feminism that we are seeing through increasing entrepreneurialism, as this Special Issue attests? We conclude this article by offering some preliminary thoughts about why it might be that this particular campaign does not fully follow such ideological framings. First, #ButNotMaternity is a digital hashtag, but it is also a feminist campaign that was run by a number of key charities and advocacy groups. In this sense, the hashtag relied on both more traditional forms of feminist organising and pressure – such as research, campaigning and applying pressure on political figures – associated with the third sector and newer tactics that we now associate with digital feminism and social media activism, such as voicing individual experiences through the hashtag. Second, the campaign did not become viral or make headline news in the same way that other digital campaigns seemed to have organically done, such as #MeToo.^4^ Instead, this was a highly coordinated campaign, making use of both new (social media) and, eventually, traditional media (newspapers). Therefore, the campaign may have been able to operate under the radar to an extent, and its lack of virality and mainstream visibility meant that it was less likely to be commercialised. This was perhaps best exemplified by a lack of a clear celebrity figure emerging as *the* spokesperson for the campaign. Conversely, therefore, this lack of virality may have acted in the campaign’s favour as it was able to be relatively effective and partially achieve its aims, which were to challenge and change lockdown maternity restrictions. Semi-popular campaigns may therefore be more effective than mainstream ones as they are not ‘hijacked’ and used to struggle over different values outside of the key goals of the campaign (Jackson and Foucault Welles, 2015; Knüpfer et al., 2022; Pegoraro et al., 2014). Though we found a few examples of ‘mumfluencers’ – those whose ‘posts are geared towards likes and encouragement’ (Wegener et al., 2022: 7) using the #ButNotMaternity hashtag – the campaign never received a large enough level of visibility to entice these enterprising mothers to take it over.

However, this is not to say that #ButNotMaternity is a perfect exemplar of feminist activism in the contemporary moment. Indeed, campaigns stemming from the third sector are by no means free of issues as feminist organisations can be imbued within the very same neoliberal logics that govern ‘freelance feminism’. Charities and women’s organisations, for example, have long been subject to insecure, tender-based and contract work, particularly in the austerity context and especially for ‘British Minority Ethnic and Refugee Women’s Organisations’ (see Bassel and Emejulu, 2018; Vacchelli et al., 2015). The austerity context that began in 2010 saw organisations having to fight for this type of work to survive, as funding that traditionally may have come from the government to support the third sector was cut, even though these same organisations were meant to step in to fill the role of the state as ‘The Big Society’ ideas were implemented (Coote, 2010). In this sense, we can see the beginnings of freelance feminism in other sites than the cultural realm some time ago. Overall, this highlights broader questions about feminist activist labour under capitalism. What kind of work gets compensated? What is a fair wage? Who should profit and what should be done with the profits? These are all long-standing questions, but which equally need to be revisited in this digital age of ‘freelance feminism’ and monetisation (see also Mendes, 2021).

Additionally, as we have discussed in the article, there was a tendency for #ButNotMaternity to centre white women and heterosexual couples. Therefore, the campaign does indeed problematically follow other forms of narrow feminist organising that are increasingly familiar within the realms of popular and neoliberal feminism (Banet-Weiser, 2018; Rottenberg, 2018). Overall, then, we can see that the entrepreneurial ethos of contemporary ‘freelance feminism’ shapes campaigns like #ButNotMaternity in complex ways. Although it may not fully follow the features of ‘freelance feminism’ in its entirety and it might show moments of pushing back against dominant norms, this does not mean that such campaigns are free from issues and do not reignite social inequalities in other ways.

## Notes

1. The Alliance comprises the following organisations: The BirthBliss Academy, Birthrights, the Birth Trauma Association, Association for Improvements in the Maternity Services, The Queer Birth Club, Make Birth Better, Paternal Mental Health, Pregnant Then Screwed, Doula UK and The Fatherhood Institute (But Not Maternity, 2022).

2. For example, for a discussion about maternity health outcomes in the Canadian context, see Shah et al. (2021).

3. There was the odd explicit exception to this rule. For example, one post advertised a talk about how COVID birth restrictions were impacting trans and non-binary people.

4. Indeed, as we touched upon previously, an initial search of mainstream news and Google News indicated that the campaign only garnered 20 news articles once duplicate and irrelevant items had been removed.
